# A kiosk survey of perception, attitudes and knowledge (PAK) of Australians concerning microbes, antibiotics, probiotics and hygiene

**DOI:** 10.1002/hpja.530

**Published:** 2021-08-30

**Authors:** Rob DeSalle, Jared Wikins, Rod Kennett

**Affiliations:** ^1^ American Museum of Natural History Sackler Institute for Comparative Genomics New York NY USA; ^2^ The National and Science Technology Center of Australia Kingston ACT Australia

## Abstract

**Issues addressed:**

To obtain a baseline of public perception, attitudes and knowledge (PAK) of Australians about microbes, antibiotics and hygiene like hand washing and use of probiotics.

**Methods:**

Using a kiosk‐based survey method at the American Museum of Natural History (AMNH), we remotely assayed PAK of Australians through their interaction with the kiosk. The surveys we used had five and seven multiple answer questions and were analysed using standard comparative approaches. We also made comparisons based on gender and on age group for many of the questions.

**Results:**

Our analyses indicate that there is a lack of general understanding of the role of microbes in everyday life among Australians. In addition, we detected some basic misunderstandings about antibiotics. While 80% of the respondents identified penicillin as an antibiotic, up to 30% of the respondents wrongly identified aspirin, Tylenol, valium and Benadryl as antibiotics. We also detected a general lack of knowledge about hand washing hygiene and probiotic use.

**Conclusions:**

Our results from around 700 Australian respondents can serve as a baseline for further PAK assessment of Australians. PAK of Australians with respect to microbes and hand washing hygiene is poor therefore public education is needed. This study should stimulate a better roadmap for public education about microbes, antibiotics, probiotics and hygiene.

**So what?:**

With the recent spread of SARS‐Cov2 and the ensuing Covid19 pandemic and the continuing rise in antimicrobial resistance, the need for assessment PAK of microbes and infectious disease has become acute.

## 1 INTRODUCTION

Public perception, attitudes and knowledge (PAK) of microbiology has been the subject of intense examination and concern in both scientific and public health circles.^1^, ^2^, ^3^, ^4^, ^5^ Generally, scientists and public health experts seek to be involved in public education about microbes, recognising that public awareness of microbes is essential for human health and well‐being.^1^, ^4^, ^6^, ^7^ A voluminous literature of studies of PAK with focus on antibiotics and antibiotic resistance exists. These studies include diverse approaches to understand public attitudes towards antibiotics in countries all across the globe.^8^ With the recent spread of SARS‐Cov2 and the ensuing Covid19 pandemic, the need for proper and immediate information for the public has become even more acute. While there are many good approaches to this public education problem, several areas of the globe have generated little information on PAK to microbes, antibiotics and hygiene.

Despite the global significance of microbes on human health,^8^, ^9^, ^10^, ^11^, ^12^, ^13^, ^14^, ^15^, ^16^, ^17^ we found few studies on Australian public attitudes towards (and understanding of) microbes and associated topics (such as antibiotics, microbial diversity, probiotics and sanitation methods). In 2015, the World Health Organization (WHO)^18^ conducted a global survey of awareness of antibiotic resistance that offered interesting conclusions about PAK in some Pacific countries but excluded Australia. Charani et al,^19^ used a small sample (n = 59) of Australian health care workers in a study assessing antibiotics stewardship and pooled their responses with other health care workers across the globe. Another Australian study on health care workers concerned a survey of medical students designed to assess their knowledge of microbiology and microbial‐related topics.^20^ The only study we are aware of that actually looked at the Australian public was conducted by Northey et al,^21^ who used a test study system to assess the efficacy of verbal education of antibiotic topics in patients using those antibiotics. This lack of perspective on the Australian public's attitudes and knowledge to microbes is striking, given that microbiology has undergone a revolution of sorts in the past decade or so^1^, ^6^, ^7^ due to the capacity to characterise communities of microbes via microbiomes.

Perhaps an excellent way to view the status of public attitudes is to examine primary and secondary teacher perceptions as these teachers need to prepare their knowledge base to be able to teach their students. At the formal and informal level of microbiology education in Australia, we note that microbiology is covered within the Australian science curriculum across several of the year levels, commencing in the primary years. However, that teachers often lack the knowledge and self‐efficacy to teach primary science and specific science content is often not covered.^22^ While there are important social health messages delivered to students (hand washing, sharing of food), the reasons for these messages is not included. Any teacher with the knowledge and confidence to cover microbes in the classroom would face additional challenges around providing practical experiences for students.

Australian schools are subject to different strict guidelines depending on their jurisdiction, the individual school and individual teachers or lab technicians. These are summarised in Science Assist: Guidelines for best practice for microbiology in Australian Schools.^23^ This creates another barrier that only the most skilled, confident and capable teacher can overcome.

While the decline of qualified secondary biology teachers is not at the same level as physics or chemistry,^24^ there are still a large number of secondary science teachers working outside of their domain.^25^ This contributes to perpetuating the lack of knowledge and practical exploration of microbes and associated topics within formal education until specialised courses are available at university.

This starting point of limited formal education in microbes and associated topics within Australia alongside disparate and disconnected public health messages and the rise of sensationalist media and social media as an influencer of peoples’ understanding of science and technology issues, is, doubtless, having an effect. However, in Australia, to date there has been little research in detailing what the general population understands about microbes and antibiotics and where their information is coming from.

In this study, we attempt to fill in part of this gap with information obtained from surveys conducted at the American Museum of Natural History (AMNH). As an informal education and research institution, the AMNH admitted prior to the Covid19 pandemic over 4.5 million visitors a year from almost every country on the planet. By placing a polling kiosk in the public areas of the museum, we were able to poll over 40,000 people of all ages and from over 170 countries. Since the sample sizes from various parts of the world are considerable for many countries (ranging from 100 to 600 respondents for about 20% of the countries other than the United States), we decided to focus on Australian understanding of microbiology using this database.^8^ The questions in the surveys were designed to test basic aspects of microbiology, antibiotics, probiotics and hygiene knowledge of the public. Survey results from Australians were compared with those from other countries selected for geographic proximity and/or commonality of language (English)’.

## 2 MATERIALS AND METHODS

As part of a larger NIH‐SEPA (US National Institutes of Health, Science Education Partnership) funded project to enhance education of the public in human microbiome topics, we developed and constructed a stand‐alone kiosk for surveying very basic aspects of public knowledge about the microbes and human health (https://www.amnh.org/research/staff‐directory/robert‐desalle under “SEPA kiosk”). Below, we describe the mechanics of the polling process and how data are collected using the kiosks.

*Polling Respondents:* In all, respondents from 171 countries participated in the two surveys on the AMNH‐SEPA kiosk over a two‐year period from December 2016‐December 2018. The response from Australian citizens for both surveys (Survey 1 = 279 and Survey 2 = 333) was substantial, lending relatively large numbers to the analysis in the present report. The demographic spread of the Australian respondents and raw numbers for the other countries we used to compare to Australia are given in Figure S1, which also shows the distribution of respondents by age and gender for Australian respondents. It also compares the Australian sample size with the non‐US (US Survey 1 = 3877 and US Survey 2 = 5916) countries included here for comparative purposes. The global sample and the US sample are shown in the inset in Figure S1. All raw data for the study are downloadable from AMNH under “SEPA kiosk.”

*Preliminary Work Driving the Polling Process:* Preliminary verbal surveys on microbes and microbiomes and observations of the behaviour of museum‐goers during 2016 guided our methodology and polling questions. For instance, this experience with kiosk behaviour indicated to us that the average visitor would at best be able to answer nine to ten questions. If three of those gathered information on gender, age and country of origin, this leaves about six to seven questions for data collection. Therefore, given the short time that people spend at kiosks answering survey questions, we focused on a short course of questions that could enlighten us about the very basic attitudes of the public on these topics. Our preliminary surveys of museum visitors indicated a very low level of understanding of microbes and especially the microbiome. In fact, the grand majority of pre‐survey visitors to the AMNH could not define what a microbiome is (data not shown). Consequently, we tailored our polling questions at a very basic level.

*The Surveys:* We conducted two surveys with four and six questions each (not including the three questions on each survey designed to collect data on age, gender and country). We posed three categories of questions in the surveys. The first category of questions attempted to address very basic knowledge of the public about microbes, antibiotics and probiotics. The second category of questions were posed to address personal habits of the public with regard to hand washing hygiene and probiotics usage. A third category consisting of a single question attempted to assess the public's confidence in their knowledge of microbes, antibiotics and probiotics.

The wording of some of the questions completely overlapped from Survey 1 to Survey 2, and some were similar between the two surveys but posed in different ways. Table 1A lists the questions asked in the first survey conducted from December 2016 to November 2017 and Table 1B lists the questions asked in the second survey conducted from December 2017 to December 2018. These tables also give the rationale for asking the questions and the information that we sought in including them in the survey. Some of the questions are objective and have discrete answers, others are subjective and were included to gain insight into the public's attitudes and habits with respect to microbes.

**TABLE 1:** Questions used in Survey 1, and Survey 2 conducted from December 2016 to November 2017 and December 2017 to November 2018 respectively

Survey 1
Q1. “Which two words come to mind when you hear the word microbe?”
1 “Germ”
2 “Disease”
3 “Tiny”
4 “Beneficial”
5 “Essential”
6 “Biodiversity”
*This question was asked to gauge the initial impressions of microbes*.
Q2.”Which of these is an antibiotic? Select as many as you like!”
1 “Aspirin”
2 “Diazepam (eg Valium)”
3 “Acetaminophen (eg Tylenol/Paracetamol)”
4 “Penicillin”
5 “Antihistamine (eg Benadryl)”
*This is an objective question with a clear correct answer – Penicillin. This question was posed to gauge the public's knowledge of antibiotics at a very basic level. If we had obtained mostly correct response to this question we planned to have proceeded in the second survey to ask a question about resistance*.
Q3.“How often do you think a person should use hand sanitiser (excluding soap)?”
1 “Frequently”
2 “Sometimes”
3 “Rarely”
4 “Never”
5 “I don't know what hand sanitizer is”
*This subjective question should give us information on some of the hygiene habits of the public. We also expected that people would know what hand sanitizer is*.
Q4.“Do you take probiotics?”
1 “I don't know what a probiotic is”
2 “Yes, regularly”
3 “Yes, but infrequently”
4 “Never”
*This question was asked as entirely an exploratory one*.
Q5.“How informed are you about the risks and benefits of antibiotics?”
1 “Knowing more about this would influence my behavior.”
2 “I am well‐informed.”
3. “This does not interest me personally.”
4. “I wish I knew more.”
*This question was asked to assess the public's confidence about the fundamental issue about antibiotics – whether there is risk as well as benefit from antibiotics. This question was.also asked* verbatim *in the second survey*.
Survey 2
Q1.“Which of the following are true statements about microbes: (check all that apply).”
1 “Microbes are too small for the naked eye to see”
2 “Microbes only have one cell.”
3 “Microbes are only in dirty places.”
4 “Microbes are essential for life.”
5 “There are many types of microbes.”
*This question was asked to follow up on questions from Survey 1 on the public's general impression of microbes*.
Q2.“For human health, microbes are:”
1 “Mostly beneficial”
2 “About half of them beneficial and half of them harmful”
3 “Mostly harmful”
4 “Have no impact on human health”
*This question was asked to gauge the public's starting point on what they think a microbe is. This question follows up on one from Survey 1*.
Q3.“How many times on a normal day do you use hand sanitizer?”
1 “0”
2 “1”
3 “2”
4 “3”
5 “4”
6 “5 or more”
7 “I don't know what hand sanitizer is”
*We hoped this subjective question would give us more precise information on some of the hygiene habits of the public over Question 3 in the first survey*.
Q4. “Which statement do you most agree with?”
1 “It is healthy to use hand sanitizer regularly in addition to soap.”
2 “Using hand sanitizer a few times a day is a healthy habit.”
3 “Hand sanitizer is a convenient e alternative to hand washing. ”
4 “Hand sanitizer should only be used when you have no other option.”
*This question was asked to gauge the public's daily use of hand sanitizer*.
Q5. “Which of these is an antibiotic? Select as many as you like!”
1 “Aspirin”
2 “Valium”
3 “Tylenol/Paracetamol”
4 “Penicillin”
5 “Benadryl”
6 “Neosporin”
7 “Azithromycin”
*This question was asked to further assess the surprising result from Survey 1, that most respondents misidentified antibiotics*.
Q6. “Do you eat or take probiotics?”
1 “Yes, every day” We expected that most of the public would
2 “Yes, a few times a month”
3 “Yes, a few times per year”
4 “Never”
5 “I don't know what a probiotic is”
*This question was asked to gauge the frequency with which the public uses probiotics, in the most general sense*.
Q7. “How informed are you about the risks and benefits of antibiotics?”
1 “Knowing more about this would influence my behavior.”
2 “I am well‐informed.”
3. “This does not interest me personally.”
4. “I wish I knew more.”
*This question was asked to assess the public's knowledge of the fundamental issue about antibiotics – whether there is risk as well as benefit from antibiotics*.

Note

Questions (Q) on Survey 1 (S1) and Survey 2 (S2). Possible answers are also shown as well as a rationale (in italics) for the question.

In some cases, answers to questions in the first survey guided the questions we asked in the second. For instance, Question 2 from the first survey (Table 1) was designed to assess whether or not there was good understanding of what an antibiotic is. If the results of the first survey had indicated an a good knowledge’ of what an antibiotic is, then we would have proceeded in the second survey to delve into antibiotic resistance. Unfortunately, as the results section will show, the public has a very poor and sometimes misleading idea of what an antibiotic is and so instead of pursuing the topic of resistance in the second survey, we attempted to verify the poor understanding of what an antibiotic is with another question focused on this basic understanding of antibiotics in the second survey.

Jones et al,^26^ and Nardi^27^ point out that composing the questions is an important aspect of survey studies. While we have based the composition of survey questions on prior knowledge of museum‐goer's behaviours, we recognise that not all survey questions are airtight. Therefore, we include a discussion of the limitations of the survey questions below and in Table 2.

**TABLE 2:** Limitations to specific questions in the surveys. S1 indicates Survey 1 and S2 indicates Survey 2, while Q1 through Q4 indicate Questions 1 through 4 on either survey (see Table 1)

Survey#/question#	Question	Limitation	Bias
S1/Q1	“Which two words come to mind when you hear the word microbe?”	Limit to two words might bias participant to pick first two words in list	Bias toward not picking beneficial or essential
S1/Q2	“Which of these is an antibiotic?"	Pick as many as you like encourages participant to pick unwanted answers	Bias toward picking more antibiotics than needed makes question less objective
S1/Q3	“How often do you think a person should use hand sanitizer (excluding soap)?”	Answer depends on participants situation	Result may not reflect attitude to hand washing
S2/Q2	“For human health, microbes are:”	By setting "half versus half" answers limited	Bias toward avoiding the response "About half of them beneficial and half of them harmful”
S2/Q4	“How many times on a normal day do you use hand sanitizer?”	Answer depends on participants situation	Result may not reflect attitude to hand washing
S2/Q5	“Which of these is an antibiotic?"	Pick as many as you like encourages participant to pick unwanted answers	Bias toward picking more antibiotics than needed makes question less objective

*The Kiosk:* The AMNH exhibitions team designed and constructed a single kiosk that could be placed in various prominent positions in the museum without clashing with design of current halls and with minimal obstruction of flow of visitors. Details on the design and construction of the kiosk are available at AMNH under “SEPA kiosk.” The kiosk used a web‐based app approach to collecting data. The web app serving the survey questions on the kiosk was built for the AMNH by Dan Melancon. This web app produces a real time archive of answers and posts a summary in several formats at least once a week. The app version we used is available upon request.

*Data Analysis:* Survey 1 was analysed separate of Survey 2 due to the difference in questions. However, in some cases, we were able to combine data from the two surveys in summary statements on particular questions. The data logs from the web app program were parsed into Excel Spreadsheets and analysed by stratifying the poll answers by country, region and continent (archived data available on request). The survey results were structured as percentage of total respondents for all comparisons. We used straightforward statistical approaches to detect statistical significance of differences between data categories.^28^

## 3 RESULTS

### 3.1 Overall survey results

**FIGURE 1:**
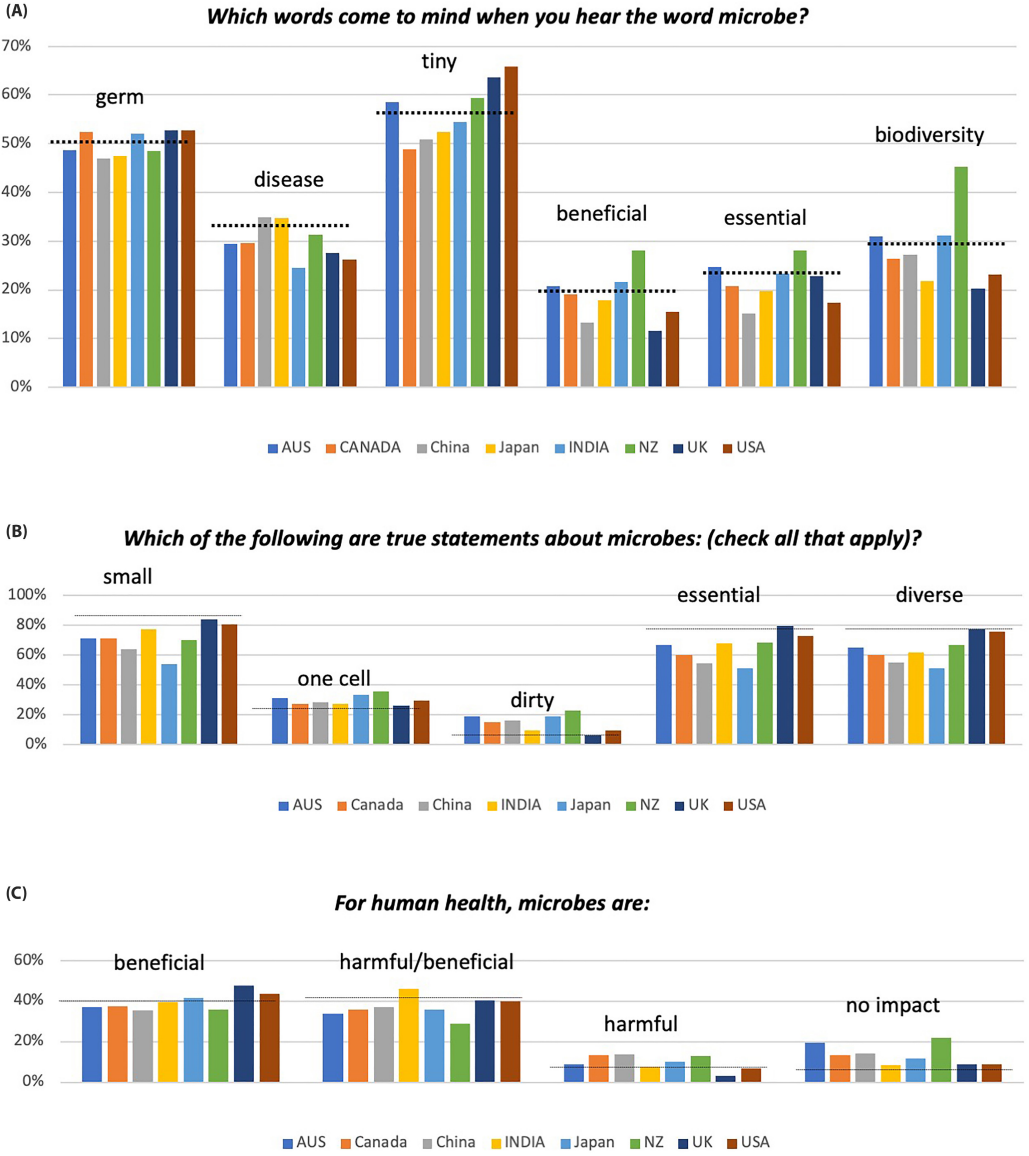
Frequency differences between Australia (AU) and the seven countries selected for comparison. Abbreviations NZ = New Zealand, US = United States, JP = Japan, IN = India, CH = China, CA = Canada and UK = United Kingdom. (A) Survey 1– Question 1, (B) Survey 2 – Question 1 and (C) Survey 1 – Question 2 (bottom) and can be referred to in Table 1. The dotted line in the figure represents the global average for that answer for the question

While there are some slight differences between Australian responses versus other countries in the study (Figure 1 through Figure 4), for each question Australian answers were not significantly different from at least two other countries for each comparison. Australian answers to all questions in the study are very similar to the global average (which includes data from 171 countries and is shown on all graphs by a dotted line). Figure S2A (Survey 1) and Figure S2B (Survey 2) show a summary of the results of the survey for the Australia and the seven countries we chose for focus.

### 3.2 General impressions about microbes

One question from Survey 1 and two from Survey 2 were designed to get a sense of the respondents’ impression and general knowledge of microbes. Figure 1 shows an example of the distribution of answers to the Survey 1 question – *“Which words come to mind when you hear the word microbe?”* For this question, we pitted three terms commonly used by people to describe microbes (tiny, disease and germ) against three positive terms that modern microbiology has discovered about microbes (essential, biodiversity and beneficial). As Figure 1 shows, “germ,” “disease” and “tiny” show up in high frequency in all countries included in this study relative to the frequency of “biodiversity,” “essential” and “beneficial.” We also analysed how Australian respondents combined words in their description of microbes. About 60% of the respondents avoid the use of the words “beneficial,” “essential” and “biodiversity” altogether when describing microbes, and only 16% avoid the use of the words “tiny,” “disease” and “germ” altogether. Thus, there is a clear preference for the use of these latter three terms. We noted a trend in answers that might explain this disparity. If an answer included the words disease or germ, there was a five times lower frequency of including any of the three words “biodiversity,” “essential” and “beneficial” in the description. However, if the word “tiny” was used, the frequency of including “biodiversity,” “essential” and “beneficial” was raised by a factor of five. These trends are very similar to those observed for the global population of respondents.

Survey 2 had two questions on it that addressed this description issue with respect to microbes. The answer profiles for these two questions are shown in Figure 1. In these questions we gave more neutral options and fewer positive options as answers. The possible answers for Question 1, Survey 2 were reduced to having only “dirty” as a negative choice, and “essential” and “diverse” as positive choices. Two neutral choices “one cell” and “small” were also allowed to be part of the response. For this survey, 65% of the Australian respondents included “essential” and “diverse” either alone or in combination with other terms in their response. Dirty was used in only about 20% of the responses and the two neutral terms were used at about the same rate as diverse and essential. These results are at odds with Survey 1, where diverse and essential were used in responses around only 40% of the time (Figure 1). This difference in answers more than likely is the result of the differences in the way the questions were posed. With this question, we could also examine association of terms. The two terms (essential and diverse) appear by themselves only 8% and 7% of the time, respectively and in combination (essential and diverse with no other terms) only 3% of the time. The use of the words “essential” and “diverse” are found in 65% of the responses and this is encouraging. These two terms are much more likely to appear in combination with small and one cell (80%), than with dirty (25%). This latter result is also encouraging, as it suggests respondents do not simply view microbes as pathogens.

For Question 2, Survey 2, we examined the usage of the word “beneficial” as a descriptor for microbes. Other choices for the question “For human health, microbes are:” with choices beneficial, half beneficial and half harmful, harmful and no impact. Only a single response was allowed. When the question is posed this way, the responses include beneficial 40% of the time, with half/half, harmful and no impact appearing 35%, 8% and 17%, respectively (Figure 1). While the “beneficial” answer is the most chosen, it is only at 40% indicating that the majority of respondents regardless of country are missing the importance of microbes in our everyday lives.

### 3.3 General impressions about antibiotics

**FIGURE 2:**
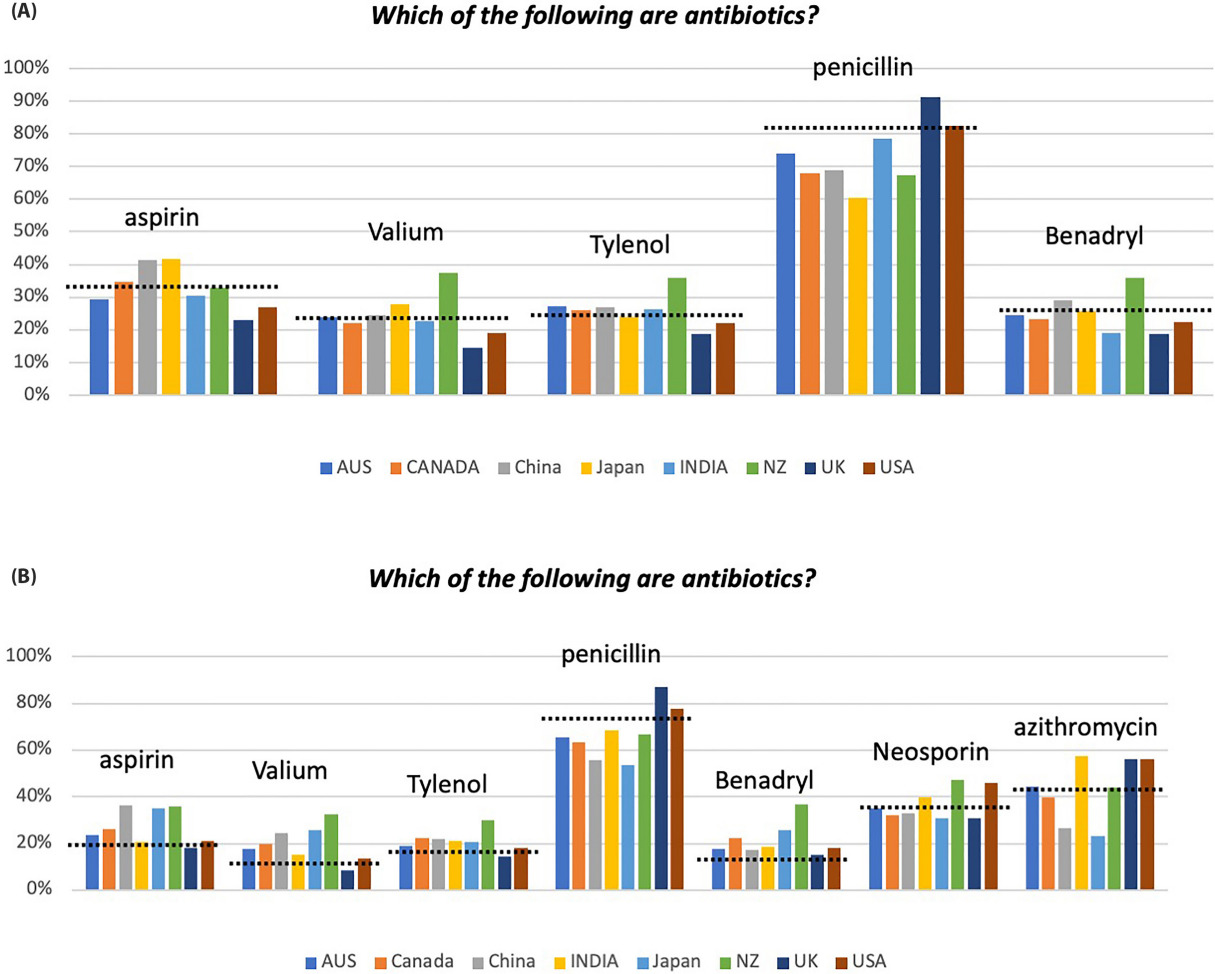
Frequency differences between Australia (AU) and the seven countries selected for comparison in reference to questions addressing knowledge of antibiotics. Abbreviations NZ = New Zealand, US = United States, JP = Japan, IN = India, CH = China, CA = Canada and UK = United Kingdom. The questions asked are (A) Survey 1 –Question 3 and (B) Survey 2 – Question 5 (bottom) and can be referred to in Table 1. The dotted line in the figure represents the global average for that answer for the question

We designed one question each for the two surveys on antibiotics. The question on Survey 1 tested for the public's knowledge of what an antibiotic is. Because the results of this question were so surprising, we designed a second question for Survey 2 on the public's capacity to identify what an antibiotic is to verify it. When asked “Which of the following are antibiotics?” and given the choices aspirin, diazepam (eg, Valium), acetaminophen (eg, Tylenol/Paracetamol), penicillin and antihistamine (eg, Benadryl), about 75% of the Australian respondents chose penicillin as one of their answers (Figure 2). However, aspirin, Tylenol, Benedryl and Valium are all chosen at 75% of the time. However, only 40% of the responses identified penicillin as the only antibiotic in the list. Even more puzzling is the incorrect identification of aspirin (7%), Tylenol (4%), Valium (6%) and Benadryl (3%) as the sole antibiotic on the list. These trends however are not unique with respect to the rest of the globe, as other countries included in this study answered at similar rates.

The second survey used a similar question but offered seven possible answers – aspirin, diazepam (eg, Valium), acetaminophen (eg, Tylenol/Paracetamol), penicillin, antihistamine (eg, Benadryl), Neosporin (actually a combination of several antibiotics) and azithromycin. Note that two antibiotics were added to the list of potential answers and that respondents could answer with as many compounds as they liked. Figure 2 and Figure S3 show the results of this survey question. Australian respondents identify penicillin as an antibiotic 65% of the time (a slight drop from the results of Survey 1), but only use the other two antibiotics on the list (Neosporin and Azithromycin) 35% and 41% of the time, respectively. These results by themselves are slightly alarming, but when we determined the rate of obtaining the correct answer for this objective question, we found that only 10% of respondents could correctly identify penicillin, Neosporin and Azithromycin alone as the antibiotics in the list of seven compounds. Australia is not alone as all other countries we included in this study display this trend of very low complete understanding of what an antibiotic is.

### 3.4 Public attitudes toward probiotics and hygiene

Our interest in this part of the survey was to obtain very basic information about the public's attitudes towards probiotics and hand washing. We asked one question about probiotics on each survey and one question in Survey 1 and two questions in Survey 2 about hand sanitiser.

**FIGURE 3:**
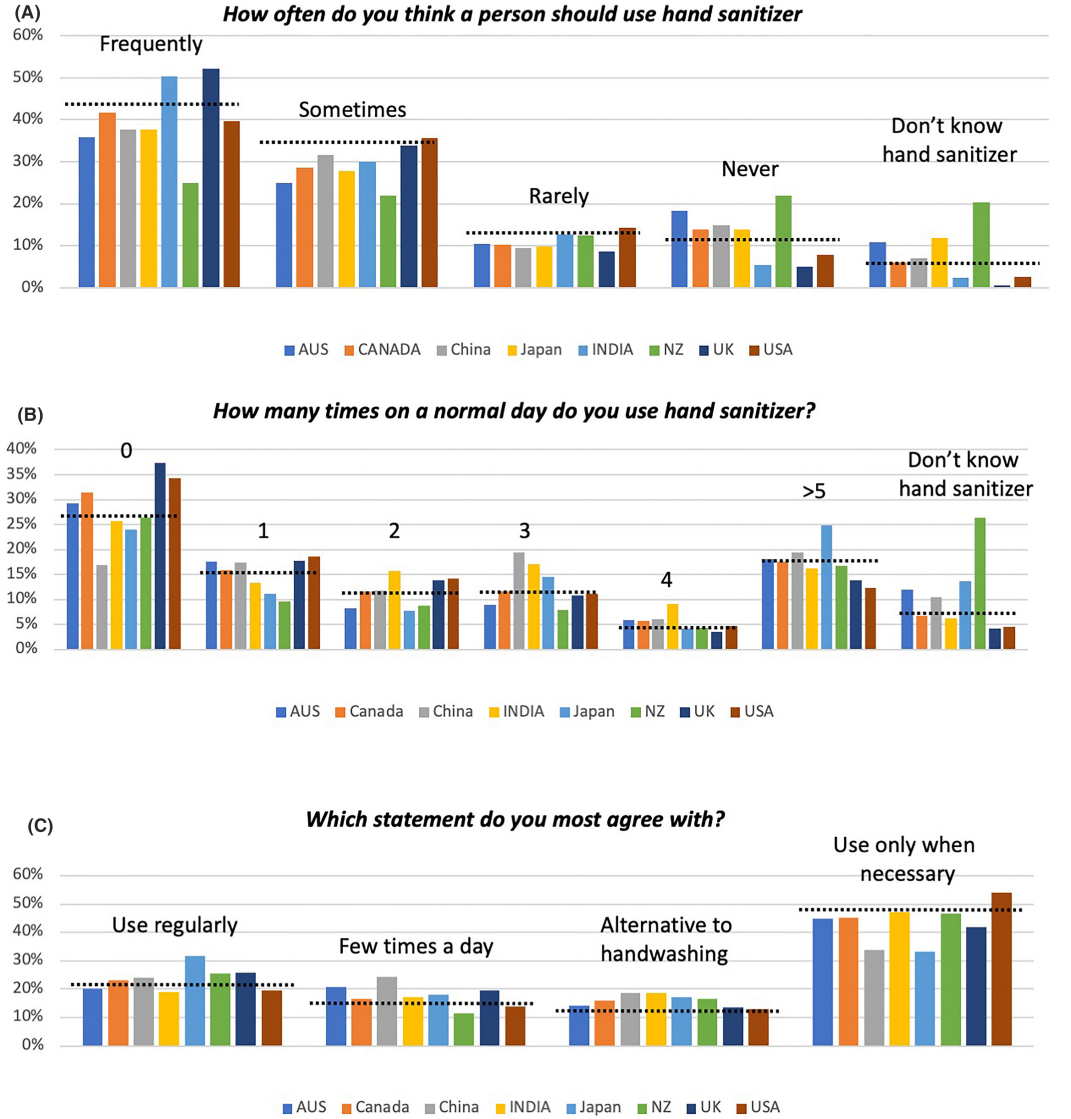
Frequency differences between Australia and the seven countries selected for comparison in reference to questions addressing use of probiotics. Abbreviations NZ = New Zealand, US = United States, JP = Japan, IN = India, CH = China, CA = Canada and UK = United Kingdom. The questions asked are (A) Survey 1 – Question 2, (B), Survey 2 – Question 3 and (C) Survey 2 – Question 4 and can be referred to in Table 1. The dotted line in the figure represents the global average for that answer for the question

*Hand washing hygiene:* One question on Survey 1 (Question 3) and two questions on survey 2 (Questions 4 and 5, Table 1) addressed issues about usage of hand hygiene products. According to analysis of the questions on Survey 1, Australian respondents answer that their use of hand sanitiser is “frequently” (33%), “sometimes” (27%) and “rarely or never” (30%) in roughly equal proportions (Figure 3). One of the Survey 2 questions were designed to give some precision to the estimate of hand sanitiser use (Figure 3). Use of hand sanitiser twice, three times and four times a day are answered in roughly equal proportions (8% each). Use of hand sanitiser once a day and greater than five times a day were answered in roughly equal frequency (17%) too. Not using hand sanitiser during the day had the highest frequency at 29%. About 11% of the Australian respondents answered that they did not know what hand sanitiser is in both surveys. When asked “*How often do you think a person should use hand sanitizer”* on Survey 2, the ranking of frequency of answers for Australian respondents was “Use only when necessary” followed by “Use regularly” followed by “Use frequently” with “Use as an alternative to handwashing” having the lowest frequency in answers (Figure 3). Australian answers do not depart from the global average or from the answers given by respondents from the other seven countries included in this analysis.

**FIGURE 4:**
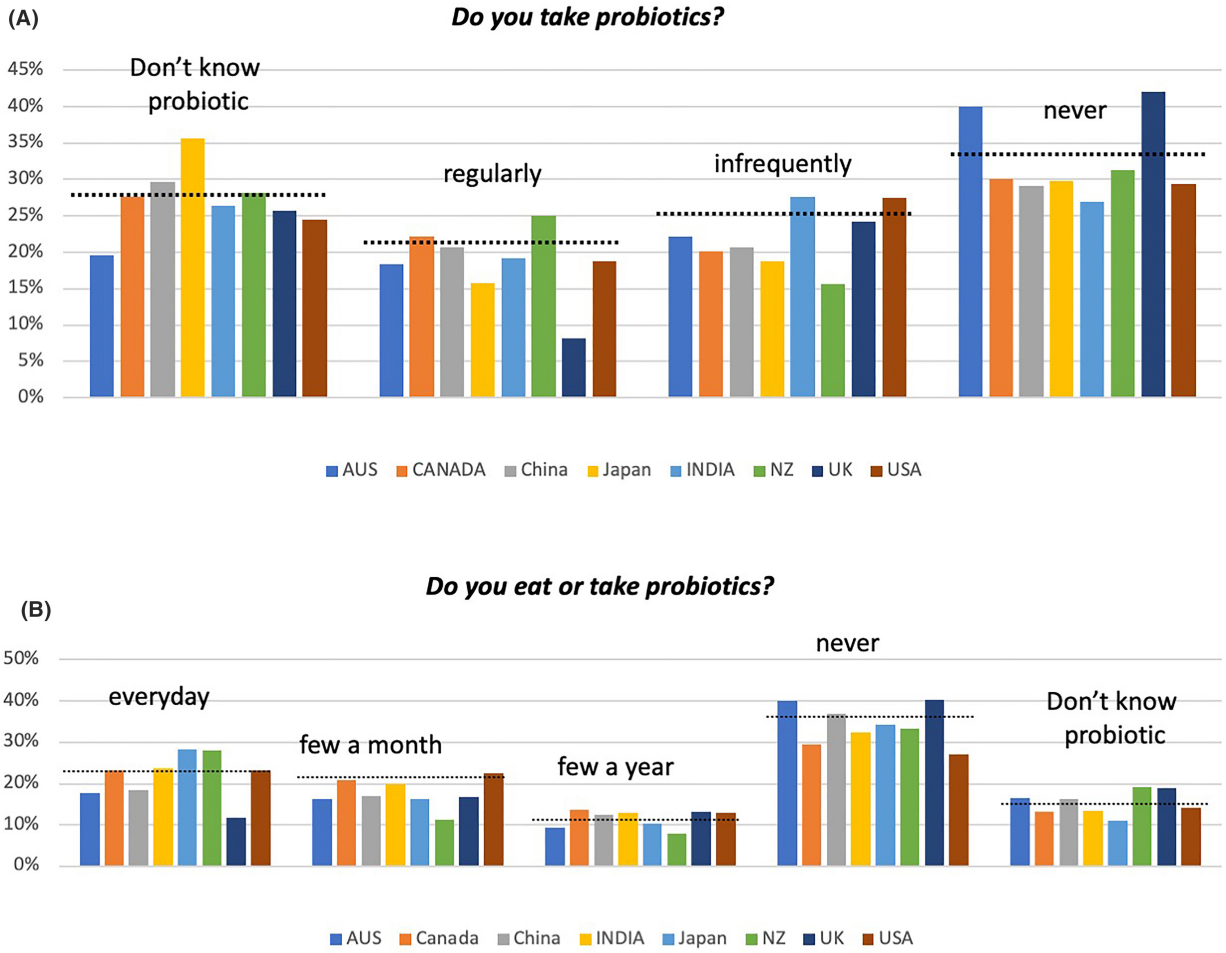
Frequency differences between Australia and the seven countries selected for comparison in reference to questions addressing use of hand sanitiser. Abbreviations NZ = New Zealand, US = United States, JP = Japan, IN = India, CH = China, CA = Canada and UK = United Kingdom. The questions asked are (A) Survey 1 – Question 4 and (B) Survey 2 – Question 6 and can be referred to in Table 1. The dotted line in the figure represents the global average for that answer for the question

*Probiotics:* One question on Survey 1 (Question 4) and one question on Survey 2 (Question 6) addressed the public's usage of probiotics. The two questions differ only in that Survey 1 attempted to get a general view of respondent's probiotic usage behaviour and Survey 2 attempted to get a more precise view of the behaviour. In the end though, we decided to combine once a month and once a year answers in Survey 2 into the infrequent category and every day into the frequent category, making the surveys in the two years comparable (frequently, infrequently and never were the three answers possible in Survey 1). The results for these questions for Australia and the seven countries included in this report are shown in Figure 4. For Australian respondents, “never” was observed with the highest frequency (40%) for both surveys followed by “infrequently” (23%) with “frequently” (18%) being the least frequent answer. Both surveys had about 18% to 20% of Australian respondents answer that they did not know what a probiotic is.

### 3.5 Confidence in knowledge

When asked to characterise their knowledge of microbes and antibiotics (*“How informed are you about the risks and benefits of antibiotics?”*) 35% to 40% of Australians feel that they are well informed and 30% to 35% think they need to know more. About 15% of Australians feel strongly that knowing more about antibiotics would influence their behaviour (Figure S4). However, nearly 20% on each survey indicated that they did not think the issue impacted them. The results from the two surveys for Australians were very similar from year to year and very similar to the responses from the seven other countries included in this study.

### 3.6 Demographic trends (gender)

**FIGURE 5:**
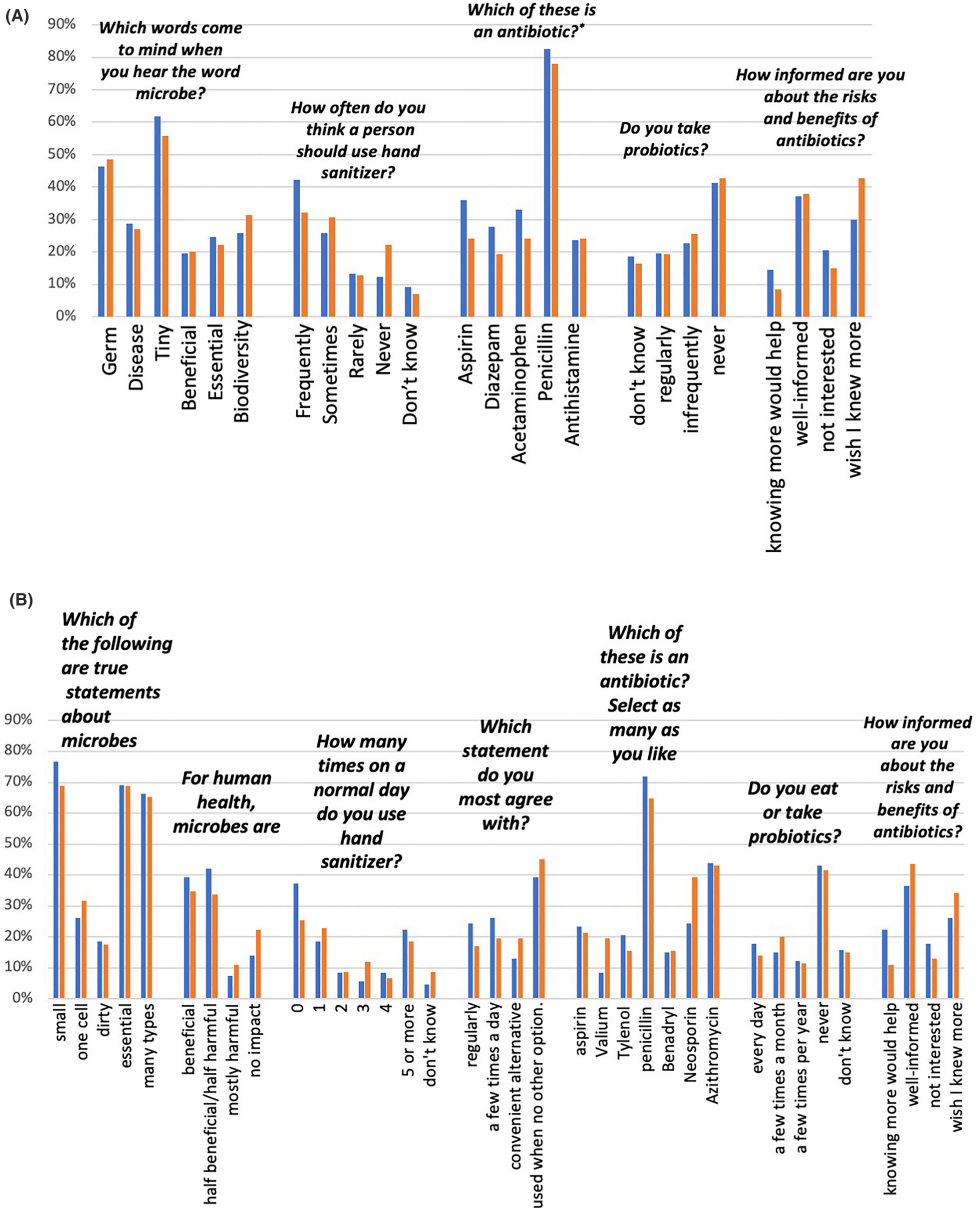
Frequency plots comparing responses from Australian males (blue) and females (orange) for both surveys. The answers are given below in the bar graphs. Questions are shown above of the distribution of answers

For Australian respondents, gender differences were slight (Figure 5). At most gender differences were about 15% between males and females. The following gender differences appear to be significant. First, with respect to confidence in knowledge about microbes and health‐related issues, males are more likely to answer knowing more would impact their behaviour and females are more likely to answer that they wished they knew more about the subject. This is an interesting way for both genders to say they would like to know more. Males are more likely to prefer the word germ than females to describe microbes and males are more likely to identify Tylenol, aspirin and valium as antibiotics than females, and females are more likely to recognise Neosporin as an antibiotic than males. Males are more likely not to use hand sanitiser (in both surveys) and also less likely to incorrectly answer that overuse of hand sanitiser is good practice.

### 3.7 Demographic trends (age)

**FIGURE 6:**
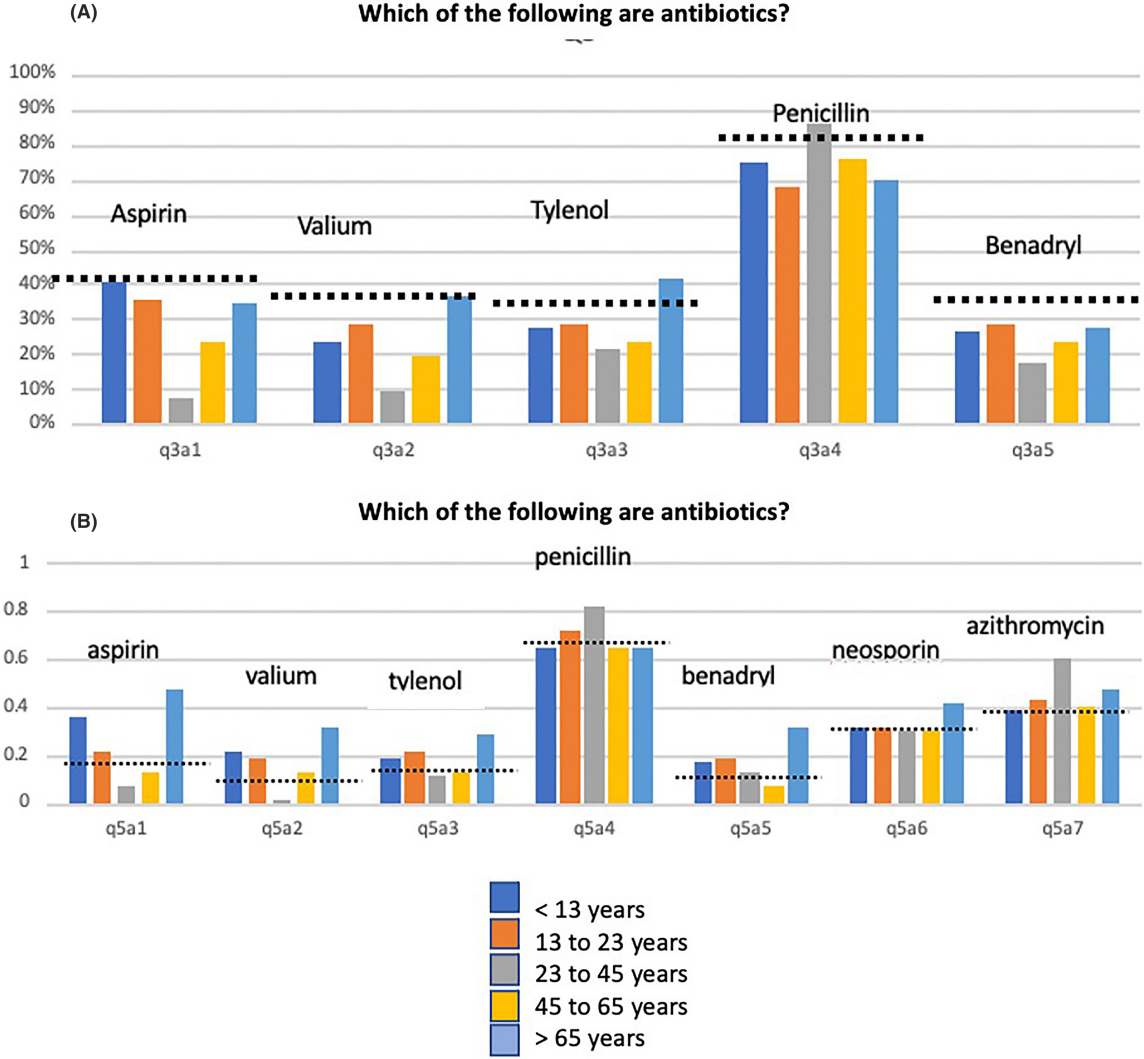
Trends in answers from Australian age categories one from Survey 1 (A) and one from Survey 2 (B). Both questions addressed antibiotic knowledge. Age categories are given on the x‐axis. The questions asked are Survey 1 – Question 3 (A) and Survey 2 – Question 5 (B) and can be referred to in Table 1. The dotted line in the figure represents the global average for that answer for the question

While many surveys avoid inferences from children (<13 years of age), we include the data for this age group in our analysis here as an initial comparative tool, with the caveat that answers from this age group are considered by many to be unreliable. The frequencies of answers across ages are remarkably similar except for a few of the answers in the survey (Figure 6). While it appears that Australians of age 65 or greater diverge from other age categories, the sample size of this age category was small. Some answers from Australian children (13 years and younger) though do tend to diverge from other age categories in several instances. The younger than age 13 category is much less likely to know what a probiotic is than other age categories. In addition, the 25‐45 age category appears to be better at discriminating antibiotics from the other compounds in our survey. It is possible that a more focused study of age differences of Australians might turn up more significant differences with respect to this subject, but our results here do not indicate a large gap between how older Australians view microbes versus younger Australians.

### 3.8 Limitations

The most important limitation to discuss, is that the participants in the survey are a biased sample as museum‐goers are recognised as a better informed and more scientifically enthusiastic segment of the general population. Several studies have evaluated the level of knowledge and perception of museum‐goers compared to nonmuseum visitors,^29^, ^30^, ^31^, ^32^, ^33^, ^34^, ^35^, ^36^, ^37^, ^38^, ^39^, ^40^, ^41^, ^42^ and have concluded that regular visitors to museums are better informed about scientific topics than nonmuseum‐goers. What this means is that the participants in the current surveys are at the more knowledgeable end of the general public PAK spectrum. The results reported here can be interpreted as a best‐case scenario with respect to the level of knowledge in regard to the survey questions. The actual PAK of the general population is more than likely substantially lower than what we report here.

Another concern is that while kiosk surveys are used to evaluate business practices and customer satisfaction, they have been used in health education and public knowledge assessment projects in a limited capacity.^43^, ^44^, ^45^ It is reasonable to suggest that kiosk interaction is similar to online surveying which has in general been considered to be very useful,^46^, ^47^, ^48^ suggesting that the results here can be useful with reservations.

Like any survey, we have attempted to overcome short comings. One problem that we anticipated but more than likely did not overcome is a language problem. This effect is most evident in the higher frequency of incorrect answers from people from Japan and China, two of the three non‐English speaking countries included in the survey. However, among the English‐speaking countries in the survey, there are some interesting results (see below). Another short coming might be caused by socio‐economic factors. Travel to the United States and the AMNH occurs in more middle and upper class families and individuals than in lower class families and so the results reported here need to be tempered with this factor in mind.

Any survey is only as good as the questions asked.^26^, ^27^ The kiosk method and online survey methods of obtaining information are limited in comparison to questions asked by in person surveys. Kiosk and online surveys need to be concise and straightforward because there is no in person questioner who can pursue an ambiguous response if given. Hence, our survey questions were designed to get general ideas of visitor responses in as concise and expedient fashion as possible. But the way these questions are posed can bias the answers and here we discuss some of the limitations shown in Table 2. There are some potential biases that arise from this scrutinising the questions that might underestimate public PAK addressed by the questions here. We point out the limitations of the questions but feel that the results of the surveys indicate a general need for better education of these topics. We did not set out to establish definitive estimates of public PAK for these issues, but rather to get an initial and general picture of PAK in Australians concerning these issues.

## 4 DISCUSSION

Over 700 Australians were surveyed through the kiosk system in place at the AMNH from December 2016 to December 2018. The survey included 22,000 respondents with demographic information from across the globe. Very basic questions about microbes including topics such as identifying antibiotics, attitudes towards microbes, probiotics and hand sanitiser were asked. Answers from Australians as well as other western countries do show some slightly significant differences with China and Japan. Language could be the reason for this difference between western and eastern countries’ patterns of answers. Australians’ answers do not diverge significantly from other western countries like the United States, Canada and geographically close New Zealand. The single outlier is the United Kingdom, whose respondents appear to answer more appropriately to questions about antibiotics (consistently avoid calling aspirin, Tylenol, Valium and Benadryl antibiotics and scoring higher on identifying penicillin as an antibiotic). However, the UK’s attitudes towards microbes in general, probiotics and hand sanitiser are not different from Australians. This pattern might suggest that the United Kingdom is doing something to educate their citizens that other western countries are not.

The EU in general and the United Kingdom in particular have focused on antibiotic resistance and educating the public about this potential global hazard. Indeed, the United Kingdom established the 2013‐2018 AMR (antimicrobial resistance) Strategy in 2013.^49^ They are currently in their second five‐year strategy plan called Tackling AMR 2019‐2024. Many antibiotic education programs in the United Kingdom are allied with the AMR strategies and one^50^ has recently reported that the UK antibiotic guardian plan (http://antibioticguardian.com/; an online registration system where participants take a pledge to properly use antibiotics) has been successful at many levels. The use of such strategies as online approaches has been recommended as a remedial measure for problems like educating the public about antibiotic resistance.^51^ The comparisons here suggest that the UK strategy works to increase knowledge about what antibiotics are.

Gender differences between Australian males and females are interesting and similar to observations made for other countries. Men differ from women in their answers in only a few cases and with about 15% difference in answers. Two examples are that men are 10% more likely to misidentify common medicines as antibiotics, and Australian men are less likely to use hand sanitiser than Australian women. Both Australian men and women want to know more about this subject with men preferring to answer that knowing more would change their behaviour. What is driving this observation is speculative but more than likely tied to child raising responsibilities, as women in Australia are more likely to be the primary household caregiver. Such a role places women closer to their children's health and hence perhaps a better understanding of antibiotics through this association. This observation appears to be prevalent among the western countries we included in the survey. On the other hand, Schröder et al^52^ have shown that worldwide, women are more likely (36%) to be prescribed antibiotics than men. This factor might suggest that women are more familiar with antibiotics than men because of this prescription pattern.

Age differences in surveys are common.^53^, ^54^ We attempted to follow current guidelines concerned with surveying children^55^ and we feel our results with respect to this age category are illuminating. In this survey, there are strong similarities of answers from different age categories. Three exceptions to this general observation are that children (less than 13 years) are less likely to know what certain terms (like *probiotic*) are, that older Australians (older than 65 years) often diverge from other age categories and that mature adults (25‐45) fare better in their understanding of microbes and antibiotics. It is well known that the accuracy of answers on objective surveys drops with age^53^ and due to smaller sample sizes of this age category, we are less confident that differences of this age category are significant. The trends for children and for young adults are believable, as these age categories have different levels of education as a result of their age and 25‐45‐year‐old Australians are more likely to have retained information from their education.

The overall picture we obtain from these surveys is that there is a deficiency in the way Australians (and other countries) perceive microbes and understand antibiotics, probiotics and hand sanitiser. The fact that given a list of five medical compounds only 40% can identify the single antibiotic (penicillin) in the list is illuminating as to the extent of knowledge of people about antibiotics. The reluctance on the part of respondents to use words like beneficial, essential and biodiverse is also telling of the public's misunderstanding of microbes and their beneficial role in human biology. The patterns we observe for use of probiotics and hand sanitiser for Australia and many other countries is also indicative of a lack of education about these important microbe‐related products.

In Australia, this lack of knowledge across the age categories is most likely established within informal learning environments– home, community, interest and age peer groups – and not consistently remedied in the formal education system. Patterns of behaviour and understanding in children are significantly influenced by the behaviours and knowledge of primary caregivers. Any successful science communication and education strategy will need to be multi‐generational and multi‐faceted to shift pre‐conceived ideas. On the positive side in a recent study of Australian's beliefs and attitudes towards science,^56^ 68.5% of respondents indicated they were “very interested” in health issues that can be addressed by science. This provides that there is more than likely a receptive audience for a well‐coordinated and communicated message.

### 4.1 Recommendations

With the limitations we have discussed previously in mind, we suggest that the study reported here can serve as a baseline for future surveys on this subject or related PAK subjects. Our survey suggests that the Australian public (and indeed the global public) need to better understand the basics of microbial life on our planet and obtain a better basic vocabulary for how we view microbes. With respect to hand sanitisers and probiotics, the Australian public needs better definitions of these items in order to facilitate better usage as our study suggests that usage of probiotics and hand sanitisers is erratic and not well understood. More programs or strategies applied in Australia like the UK five‐year AMR strategy approach are needed in order to bring the public up to speed on the advances that have been made in the past decade with respect to microbes and microbiomes. However, we suggest that the entry point for where this education begins needs to be reassessed and adjusted to the public's general knowledge of microbes, antibiotics, probiotics and other microbe‐related concepts.

## CONFLICT OF INTEREST

The authors declare that they have no conflicts of interest.

## Supporting information

Fig S1

Fig S2A

Fig S2B

Fig S3

Fig S4
