# Supplementary figures and images for: A kiosk survey of perception, attitudes and knowledge (PAK) of Australians concerning microbes, antibiotics, probiotics and hygiene
Source: Health Promot J Austr. 2021 Aug 30;33(3):838–51. doi: 10.1002/hpja.530 (PMC8847535; doi:10.1002/hpja.530)

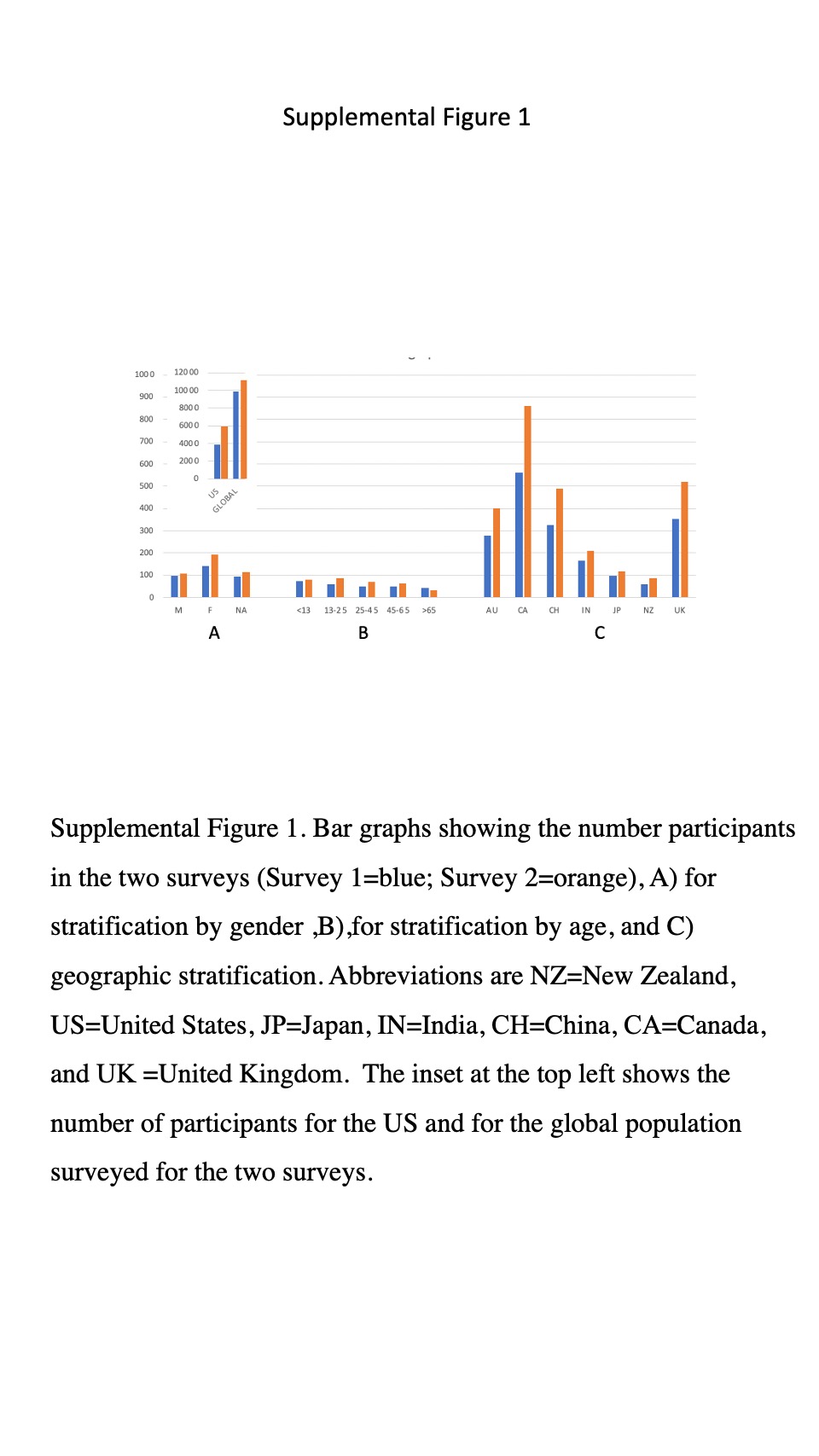

Supplement: Supplementary file 1 — Fig S1 [file HPJA-33-838-s003.jpeg]

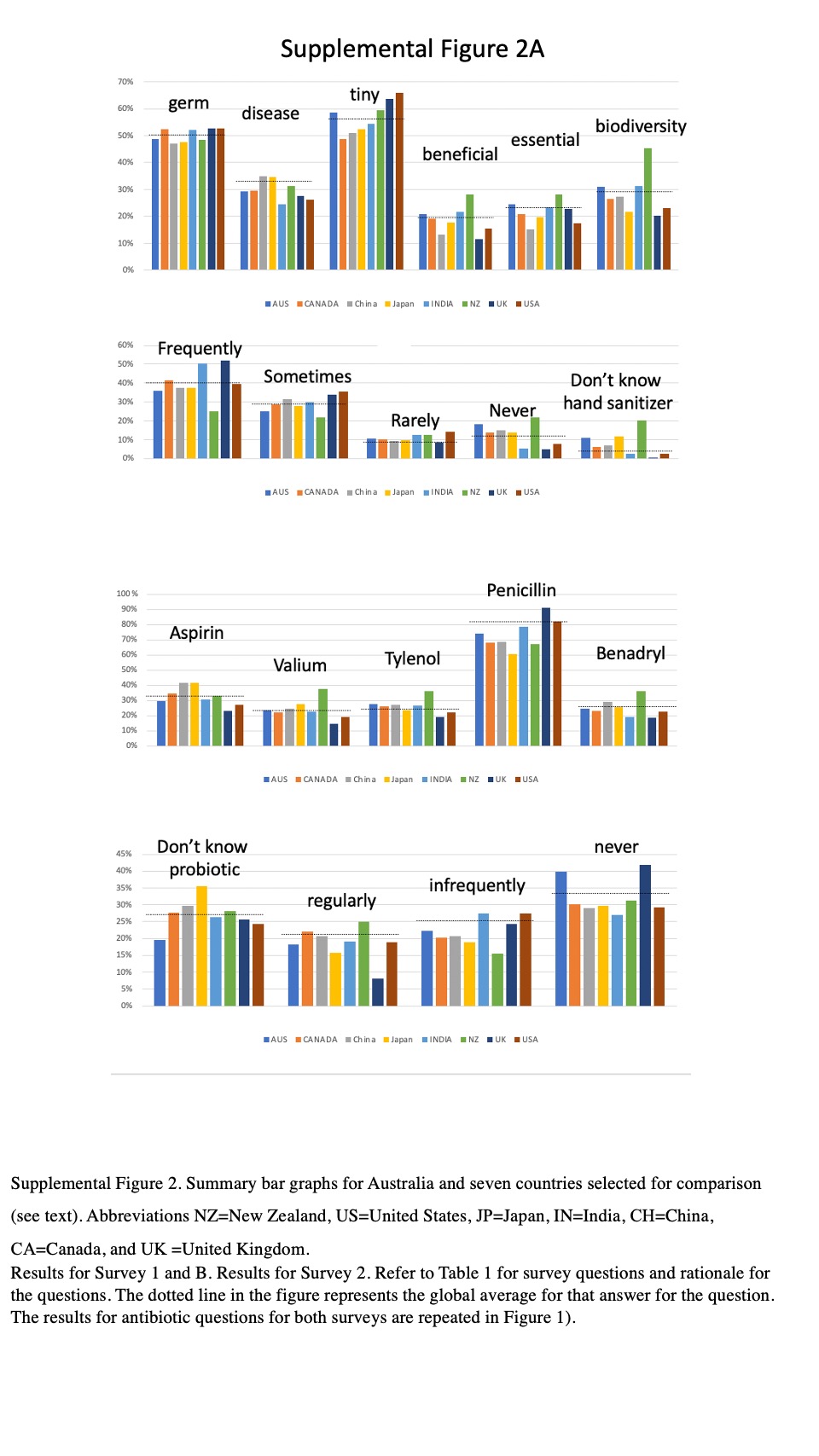

Supplement: Supplementary file 2 — Fig S2A [file HPJA-33-838-s001.jpeg]

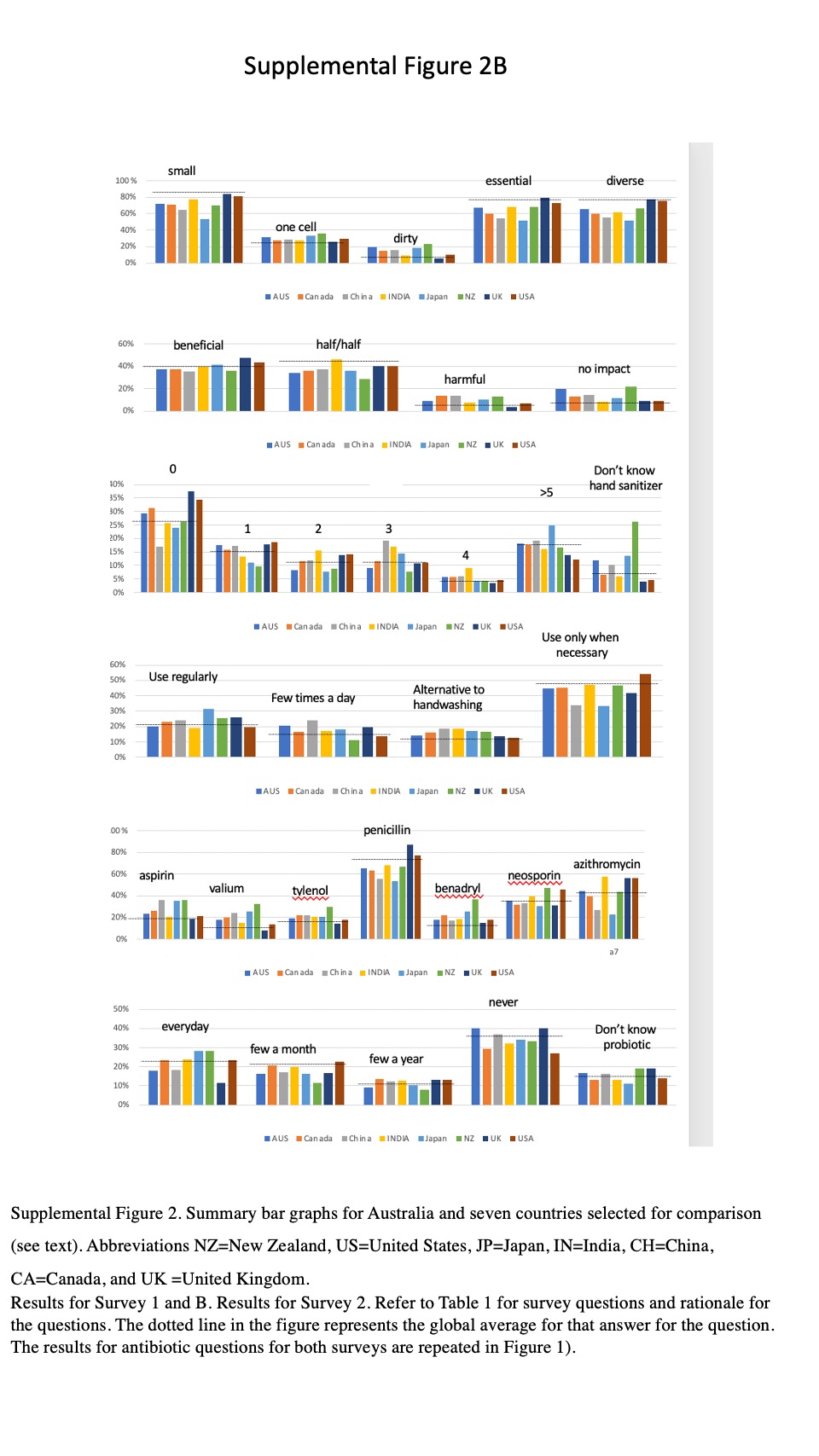

Supplement: Supplementary file 3 — Fig S2B [file HPJA-33-838-s005.jpeg]

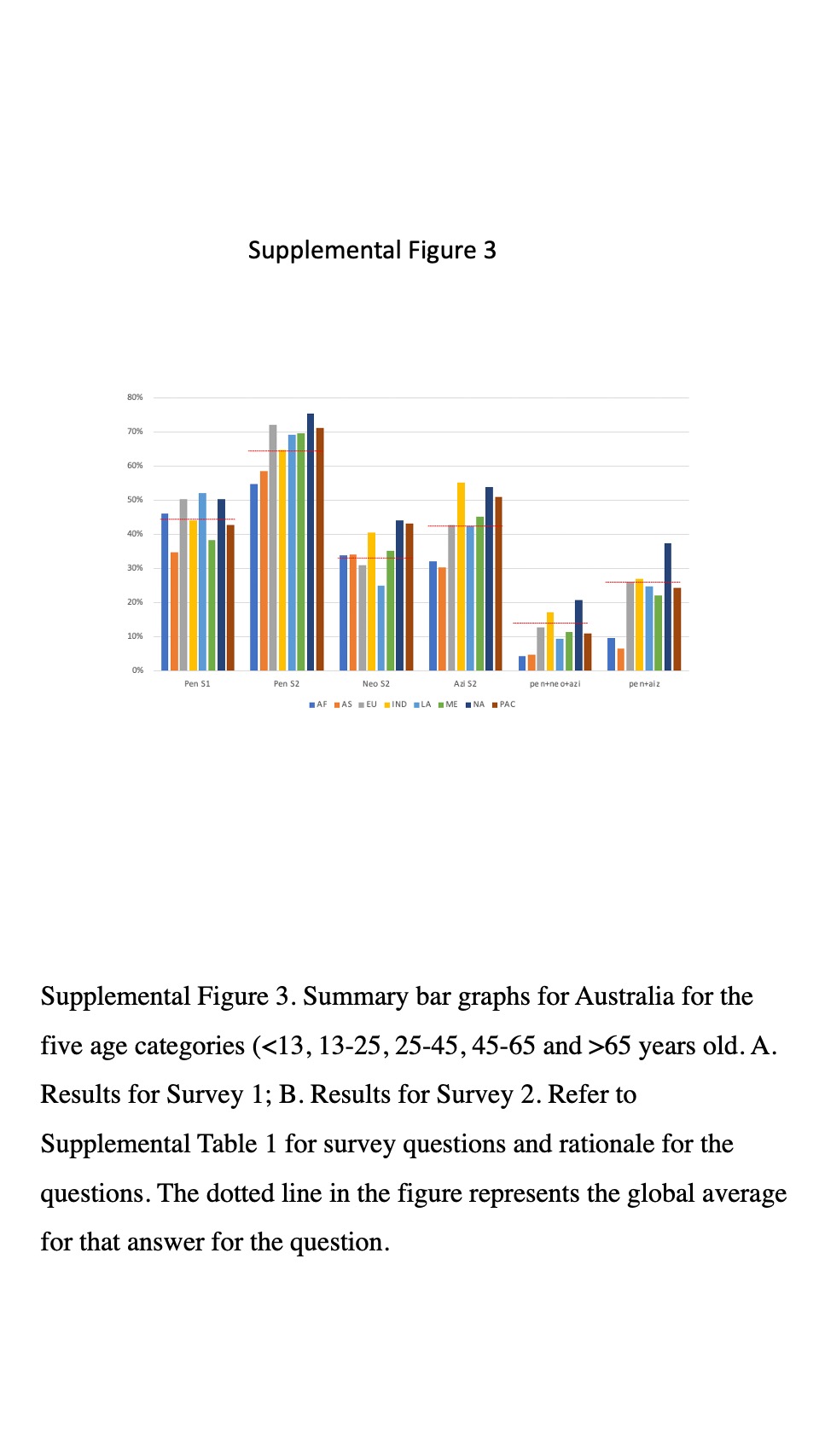

Supplement: Supplementary file 4 — Fig S3 [file HPJA-33-838-s002.jpeg]

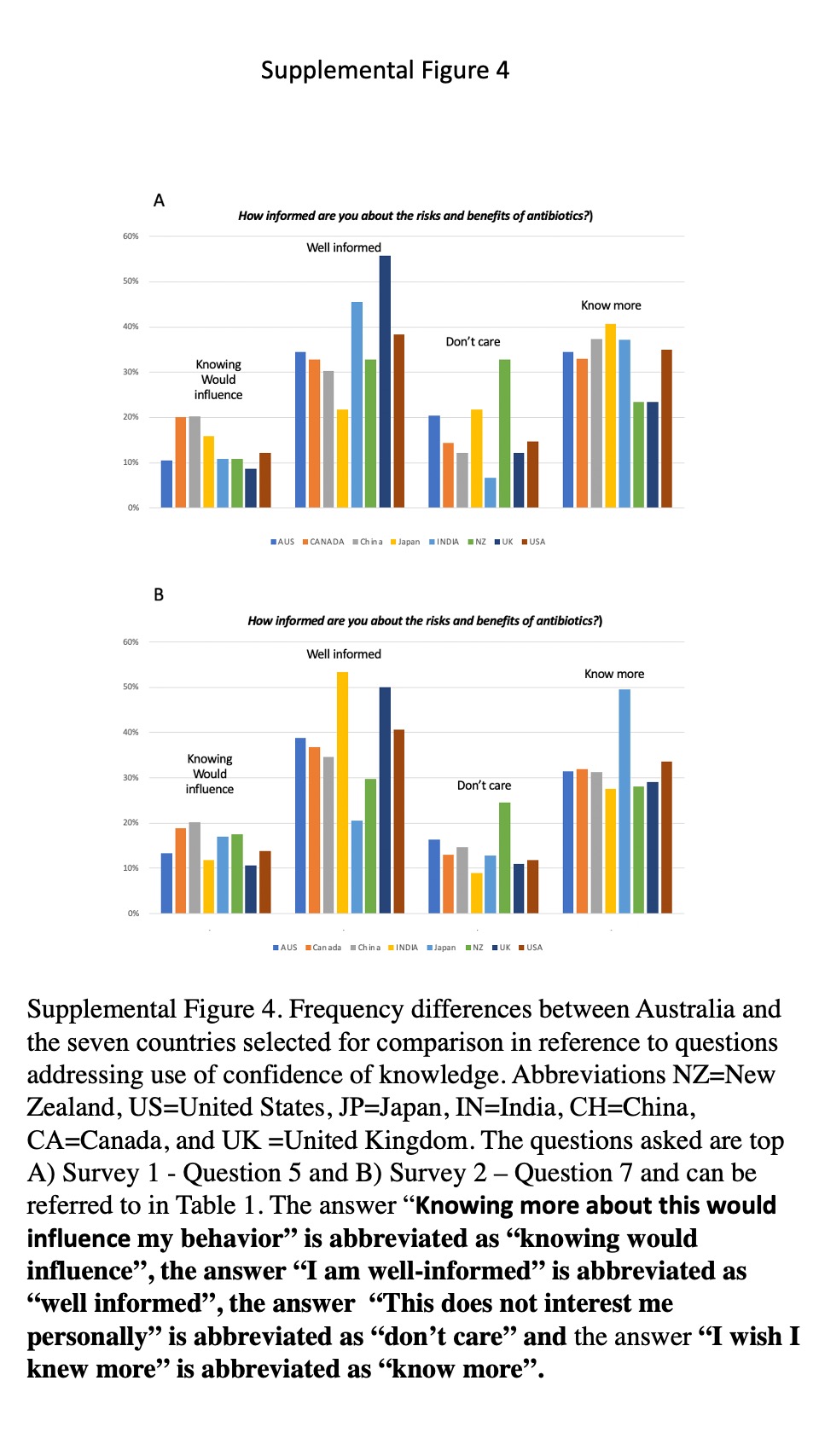

Supplement: Supplementary file 5 — Fig S4 [file HPJA-33-838-s004.jpg]
